# Simultaneous quantification method for eleutheroside B, eleutheroside E, chiisanoside, and sesamin using reverse-phase high-performance liquid chromatography coupled with ultraviolet detection and integrated pulsed amperometric detection

**DOI:** 10.1016/j.heliyon.2022.e12684

**Published:** 2023-01-03

**Authors:** YeaIn Kim, Young-Ju Jung, Hae-Jee Yoon, Ha-Jeong Kwon, Seon-Pyo Hong

**Affiliations:** a Department of Oriental Pharmaceutical Sciences, Graduate School, Kyung Hee University, 26 Kyungheedae-ro, Dongdaemun-gu, Seoul, 02447, Republic of Korea; b Department of Oriental Pharmaceutical Sciences, College of Pharmacy and Kyung Hee East-West Pharmaceutical Research Institute, Kyung Hee University, 26 Kyungheedae-ro, Dongdaemun-gu, Seoul, 02447, Republic of Korea; c Biometrology Group, Division of Chemical and Biological Metrology, Korea Research Institute of Standards and Science, 267 Gajeong-ro, Yuseong-gu, Daejeon, 34113, Republic of Korea

**Keywords:** RP-HPLC-UV-IPAD, Eleutherosides, Chiisanoside, Sesamin, High sensitivity, integrated Pulsed amperometric detection

## Abstract

We developed a method combining ultraviolet (UV) detection and integrated pulsed amperometric detection (IPAD) to simultaneously analyze eleutheroside B, eleutheroside E, chiisanoside, and sesamin. The gradient elution system allowed complete separation of all target components within 35 min, and showed limits of detection of 0.006–0.020 μg/mL and limits of quantification of 0.018–0.050 μg/mL. The linear regression coefficients of determination were 0.9990–0.9998. All inter− and intra-day precision values were below 4.89%, and the average recoveries were 97.79–104.40%. The developed approach exhibits excellent reproducibility, sensitivity, and selectivity without requiring any complicated pre-treatment, and is therefore expected to be helpful as a tool for establishing appropriate content criteria for *Acanthopanax* species.

## 1 Introduction

*Acanthopanax* root bark refers to the bark of the roots and stems of *Acanthopanax sessiliflorum seeman* or other homogeneous plants in the Araliaceae family [1]. *Acanthopanax* root bark is used in oriental medicine [2] for various symptoms, such as quadriplegia, convulsions of the hands and feet, weakness of the waist, knee, and lower leg, fractures, bruises, and edema, and exhibits immune enhancement [3], anti-inflammatory [4], anti-allergenic [4], and hepatotoxicity inhibition [5] effects. *Acanthopanax* root bark contains lignans such as eleutheroside B, eleutheroside E, and sesamin [6,7] as well as other components including chiisanoside and ciwujianoside [[8], [9], [10]]. Among them, eleutherosides B and E have high antioxidant activity and nitrite removal effects [11], and chiisanoside has been shown to prevent osteomalacia [12]. Sesamin has a growth inhibition effect on KATO III cells and an apoptosis-inducing effect [13]. The *Acanthopanax* genus contains dozens of species, and in order to use the different species appropriately according to their efficacy, the contents of the above four components must be measured. For this, a new analysis method capable of simultaneously analyzing the four components is required.

Previous studies have analyzed eleutheroside B, eleutheroside E, chiisanoside, and sesamin with various techniques including high-performance liquid chromatography (HPLC)-ultraviolet (UV) [14,15], HPLC-diode array detection (DAD) [16], gas chromatography (GC)-mass spectrometry (MS) [17], and HPLC-electrospray ionization (ESI)-MS [18,19]. Of these, HPLC-UV and HPLC-DAD are the most commonly used analytical methods, but these approaches offer poor sensitivity for components with weak chromophores. On the other hand, while the HPLC-MS and GC-MS methods are highly sensitive, they are not commonly used due to the cost and equipment required.

One technique for the detection of sugars and amines is pulsed amperometric detection (PAD) [[20], [21], [22]]. In PAD, current is measured at one potential for a set period of time and integrated to obtain the charge. One variant of PAD is integrated PAD (IPAD) [22], in which current is measured at more than one potential and is continuously integrated throughout the cycle. Sugar analysis has been achieved through high-performance anion exchange chromatography (HPAEC) coupled with IPAD (HPAEC-IPAD) based on its high selectivity to sugars. However, the HPAEC-IPAD method has rarely been used for glycosides due to its low separation capability.

For eleutheroside B, eleutheroside E, and chiisanoside, the IPAD method is more sensitive than the UV method, while conversely, the UV method is more sensitive to sesamin. For the simultaneous and accurate analysis of these four components, we developed a reversed-phase (RP)-HPLC-UV-IPAD method that combines UV and IPAD techniques. Here, we present the pre-treatment and analysis of the four above-mentioned components contained in the root bark of various *Acanthopanax* species using the developed RP-HPLC-UV-IPAD method.

## 2 Experimental

### 2.1 Materials

Chiisanoside was purchased from Wuhan ChemFaces (Wuhan, Hubei, China). Eleutheroside B, eleutheroside E, and sesamin were purchased from ChromaDex (Santa Ana, CA, USA). Solutions of 50% sodium hydroxide and acetonitrile (HPLC grade) were obtained from Fisher Scientific (Fairlawn, NJ, USA). Salicin was purchased from Merck (Darmstadt, Germany). Purified water (18 MΩ-cm) was used for the mobile phase. *Acanthopanax* species were provided by Professor Chang-Soo Yook.

### 2.2 RP-HPLC-UV-IPAD

The IPAD system (Dionex, Sunnyvale, CA, USA) was made up of a Au flow cell and a solvent-compatible cell. The waveforms (six-potential) were as follows: E1 = +0.1300 V (from 0.0000 to 0.0400 s); E2 = +0.3300 V (from 0.0500 to 0.2100 s); E3 = +0.5500 V (from 0.2200 to 0.4600 s); E4 = +0.3300 V (from 0.4700 to 0.5600 s); E5 = −1.6700 V (from 0.5700 to 0.5800 s); and E6 = +0.9300 V (0.5900 s). The IPAD data were controlled via the Chromeleon program (Dionex). The HPLC equipment, consisting of a Model Nanospace SI-2**/**UV detector, column oven, and dual pump, was purchased from Shiseido (Tokyo, Japan). The data from the UV detector (set to 210 nm) were analyzed with the dsChrom program (Donam Instrument, Seoul, Korea). The columns used were an Eclipse Plus C-18 column (150.00 × 2.10 mm I.D.; 3.50 μm, Agilent, Santa Clara, CA, USA) and an Eclipse Plus C-18 guard column (12.50 × 2.10 mm I.D.; 5.00 μm, Agilent). The mobile phase was composed of solvent A [acetonitrile: pure water (0:10)] and solvent B [acetonitrile: pure water (8:2)]. The following gradient conditions were applied: 10.0% B (0–6.0 min), 10.0–15.0% B (6.0–7.0 min), 15.0% B (7.0–10.0 min), 15.0–50.0% B (10.0–25.0 min), 50.0–75.0% B (25.0–26.0 min), and maintenance at 75.0% B (26.0–35.0 min). The mobile phase, a mixture of water with acetonitrile as above, was made on a daily basis, degassed by vacuum filtration, and then sonicated for 20 min before use. The post-column eluent was 200 mM NaOH supplied at a flow rate of 0.8 mL/min using a single pump (Shiseido Nanospace SI-2) with pump heads made of metal-free polyether ether ketone (PEEK) resin, which is compatible with alkaline solvents. The 200 mM NaOH was made *via* vacuum filtration on a daily basis and purged with helium throughout the experiment to minimize carbon dioxide absorption.

### 2.3 Preparation of standard solutions

The sesamin stock solution was produced by adding 1 mg sesamin and 1 mL dimethyl sulfoxide to an Eppendorf tube. The stock solutions of eleutheroside B, eleutheroside E, and chiisanoside were produced by adding 1 mg of each standard and 1 mL pure water to Eppendorf tubes. The stock solutions were diluted to a suitable concentration range. The salicin (IS.) per sample was 10.0 μg/mL.

### 2.4 Preparation of sample solutions

The following *Acanthopanax* species were used [23]: *Acanthopanax divaricatus* var*. albeofructus* (ADA)*, A. divaricatus f. flavi-flos* (ADF)*, A. sieboldianum* (ASB)*, A. divaricatus f. distigmatis* (ADD)*, A. gracillstylus* (AG)*, A. koreanum* (AK)*, A. senticosus* (ASC)*, A. senticosus* var*. subinermis* (ASS)*, A. trifoliatus* var. *setosus* (ATS), and *A. sessiliflorum* (ASF). Bark was first collected from the 10 *Acanthopanax* species, and then the inner parts of the bark were removed and only the outer parts were used. The treated bark was ground with a blender and sifted through a 355-μm sieve (No. 45), and only the powder was retained. One gram of each sample was extracted using 20 mL of 70% methanol (MeOH) via 60 min of reflux extraction and then filtered. The filtrate (0.1 mL) in an Eppendorf tube was dried using a SpeedVac. Pure water and salicin (I·S.) solution were added to the dried samples. One milliliter of the final solution contained 10 μg of salicin and 10 mg of bark powder.

### 2.5 Method validation

Standard calibration curves were developed for all components. The regression equation *y = ax + b* was used, where *x* and *y* are the sample mass and sample peak area ratio (component/internal standard), respectively. Evaluation of the analytical precision was conducted through inter- and intra-day assays. Four injections ([eleutheroside B, 0.18; eleutheroside E, 0.25; chiisanoside, 0.35; sesamin 0.50], 100.0, 200.0, and 500.0 ng) were conducted three times a day for five consecutive days. Recovery tests for eleutheroside B, eleutheroside E, chiisanoside, and sesamin were conducted by comparing each standard (100.0, 200.0, and 500.0 ng) to the ASF root bark extract samples. Each sample was measured by performing three injections.

## 3 Results and discussion

### 3.1 Systematization of the RP-HPLC-UV-IPAD method

**Fig. 1:**
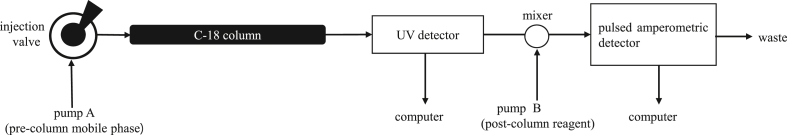
Schematic diagram of the RP-HPLC-UV-IPAD method.

PAD is an electrochemical method that measures the positive potential produced by carbohydrate oxidation on a gold electrode, enabling the direct analysis of carbohydrates at low pico-mol levels [24]. Protones in the sugar part of glycosides dissociate under alkaline conditions (NaOH) (pH > 11) and become electrochemically active. Therefore, it is essential to supply a NaOH solution in PAD for the detection of glycosides. Fig. 1 shows a schematic diagram of the RP-HPLC-UV-IPAD method. The eluent passes through the column and UV detector, is blended with the NaOH solution by a mixer, and then flows into the IPAD. In this work, a C-18 column was used to separate the target components. However, since the binding group of the C-18 column is decomposed in basic conditions, the NaOH solution was used as a post-column reagent. In our previous paper [25], the sensitivity of PAD was examined according to the flow rate and concentration of the NaOH solution; 0.8 mL/min was found to be the optimum flow rate for the NaOH solution. When the flow rate was less than 0.8 mL/min, the S/N ratio rapidly decreased. At a flow rate of 0.8 mL/min, the PAD signal was significantly improved in the range of 100–200 mM NaOH. While carbonate ions produced by absorbing carbon dioxide in the NaOH eluent can reduce the retention time as a pushing agent in anion exchange chromatography, in the current case, the carbonate ions in the post-column NaOH eluent did not significantly affect the retention time or the chromatographic properties.

Previous studies have shown that the IPAD method had good sensitivity for eleutheroside B, eleutheroside E, and chiisanoside, but not for sesamin. In contrast, the UV method has demonstrated good sensitivity for sesamin but not for the other components. Accordingly, eleutheroside B, eleutheroside E, and chiisanoside were determined with IPAD and sesamin was determined with UV in this work. Three different types of waveforms are available for PAD: triple-potential, quadruple-potential, and six-potential. The triple-potential waveform has the disadvantage of a slow loss of gold from the working electrode surface as a result of having a positive cleaning potential. The quadruple-potential waveform has better reproducibility than the triple-potential waveform since the products resulting from the oxidation of carbohydrates can be removed from the electrode at high negative potentials. The developed six-potential waveform is used for amino acid or amino sugar analysis. In our previous paper [25], we tested these three types of waveforms and found the six-potential waveform to be the most sensitive; therefore, it was selected for use in the current study. The adopted waveform offers a reproducible response through the use of a negative cleaning potential [26], but there was still a concern about contamination of the gold electrode by the acetonitrile in the mobile phase of the RP-HPLC-UV-IPAD system. IPAD sensitivity is known to be reduced by large volumes of acetonitrile [25]. Accordingly, a 2.1 mm-diameter C-18 column was used at a flow rate of 0.20 mL**/**min to decrease the amount of acetonitrile mixed with NaOH. Also, to maintain sensitivity, the gold electrode was cleaned by rubbing the surface with a pink eraser (P/N. 049721) once *per* week and by polishing with a polishing kit (P/N. 036313) every two months. Slight changes in sensitivity were sufficiently corrected with the use of internal standards.

### 3.2 Optimized extraction solvent and method

We examined the extraction efficiency of the target components in *Acanthopanax* root bark using ethanol (EtOH), MeOH, and water, and found that MeOH was more effective for extraction than EtOH and water. We next examined various concentrations of MeOH, namely 40–80% MeOH, and found 70% MeOH to show the best extraction efficiency [Supplemental Fig. 1(A)].

Sonication and reflux extraction methods using 70% MeOH as the extraction solvent were then evaluated with extraction times of 30, 60, and 90 min. The reflux method showed better extraction efficiency compared with the sonication method. In terms of time, the reflux method showed the best extraction efficiency at 60 min of extraction. Thus, we selected the 60-min reflux extraction method [Supplemental Fig. 1(B)].

### 3.3 Method validation

#### 3.3.1 Sensitivity and linearity

Table 1 shows the results of linearity and sensitivity for the four target components. The linear regression coefficients of determination were 0.9990–0.9998, indicating good linearity.

**Table 1:** Linear range, linear equation, correlation coefficient, and limits of detection and quantification for the target components.

Compound	Linear range (μg/mL)	Linear equation	R2	UV		IPAD		UV	
				LOD (μg/mL)	LOQ (μg/mL)	LOD (μg/mL)	LOQ (μg/mL)	LOD (μg/mL)	LOQ (μg/mL)
eleutheroside B	0.018–50.00	y = 0.0357x + 0.0064	0.9998	0.030	0.090	0.006	0.018	0.19^a^	0.55^a^
eleutheroside E	0.025–50.00	y = 0.0811x + 0.0489	0.9993	0.020	0.055	0.008	0.025	0.25^a^	0.71^a^
Chiisanoside	0.035–50.00	y = 0.0017x − 0.0007	0.9990	0.100	0.300	0.010	0.035	0.20^b^	–
Sesamin	0.050–50.00	y = 0.0032x − 0.0006	0.9996	0.020	0.050	1.650	5.000	0.40^c^	1.2^c^

a Data from J. Pharm. Biomed. Anal. 2011, 55, 908-915.

b Data from Nat. Prod. Sci. 2003, 9, 45-48.

c Data from Food Chem. 2019, 281, 140-146.

**Fig. 2:**
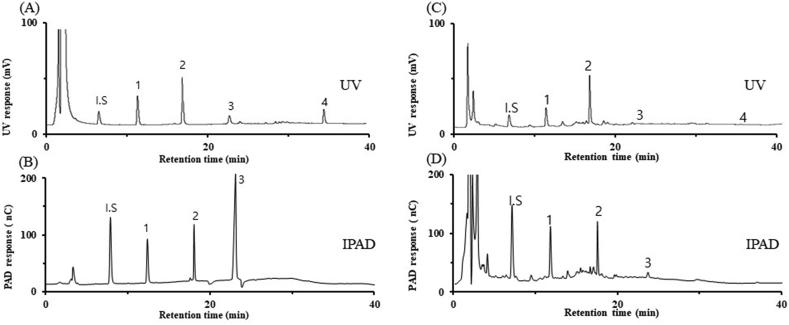
Chromatograms of UV and IPAD using standard components (A and B) and *Acanthopanax sessiliflorum* root bark (C and D). Peaks: I·S., salicin; 1, eleutheroside B; 2, eleutheroside E; 3, chiisanoside; 4, sesamin.

The limits of quantification (LOQs) are listed in Table 1. For UV, the LOQs were 0.300, 0.055, 0.090, and 0.050 μg/mL for chiisanoside, eleutheroside E, eleutheroside B, and sesamin, respectively. For IPAD, the LOQs were 0.035, 0.018, and 0.025 μg/mL for chiisanoside, eleutheroside B, and eleutheroside E, respectively. Based on the results, the LOQs for the IPAD method for chiisanoside, eleutheroside B, and eleutheroside E were 2.2–8.6 times higher than those of the UV method. As previously mentioned, sesamin was detected only using the UV method. Our UV method showed 2.0–20 times higher sensitivity in detecting the four components than other existing methods [[27], [28], [29]]. Our new RP-HPLC-UV-IPAD method enabled the simultaneous analysis of the four target components by combining UV detection [for sesamin, Fig. 2(A)] with IPAD detection [for chiisanoside, eleutheroside E, and eleutheroside B, Fig. 2(B)]. The developed technique also exhibited sufficient sensitivity to quantify trace amounts of the four target components from the root bark of various *Acanthopanax* species.

#### 3.3.2 Accuracy and precision

Inter- and intra-day precision was calculated by quantifying samples three times a day for five days. The range of relative standard deviation (RSD) was 0.34–4.89% in the inter-day assay and 0.03–2.93% in the intra-day assay (Supplemental Table 1). Accuracy evaluation of this method was performed through recovery testing (Supplemental Table 2). The RSD range and mean recovery were 1.36–4.42% and 97.79–104.40%, respectively, for ASF root bark extract. These results indicate that the developed method has good precision and accuracy.

### 3.4 Analysis of the root bark of Acanthopanax species

The isocratic elution method was found to require long analysis times, so a gradient elution method was used. The four components in the root bark of various *Acanthopanax* species were analyzed within 35 min through our method [Fig. 2(C) and (D)]. The amounts of the components in each tested *Acanthopanax* species are listed in Table 2. Regarding eleutheroside B, the amount in ASC (82.76 ± 0.04 μg/g) was 28–129 times higher than in the other nine species (0.64 ± 0.03–3.00 ± 0.04 μg/g). Regarding eleutheroside E, the amounts in ADA, ADD, ASF, and ASC were relatively high (73.89 ± 0.07–134.37 ± 1.43 μg/g), but the levels in other species were relatively low (1.94 ± 0.01–20.86 ± 0.18 μg/g). Regarding chiisanoside, the levels in ASS (185.08 ± 7.49 μg/g) and ASB (111.93 ± 1.40 μg/g) were 2.7–16 times higher than those of other species (11.42 ± 0.35–41.30 ± 0.52 μg/g). Regarding sesamin, the amounts in ADF (28.20 ± 1.39 μg/g), AK (21.84 ± 0.18 μg/g), and ASF (20.91 ± 0.95 μg/g) were high, while other species had lower content. No species had a high content of all four components. These findings indicate the need to select the particular species for use according to the desired efficacy. If high antioxidant activity is required, ASC, which has high eleutheroside B content, or ADA, which has high eleutheroside E content, would be suitable. If protective action against osteomalacia is sought, ASS, which has high chiisanoside content, would be the most suitable. In this way, the developed analytical method may be used as a tool for establishing appropriate content criteria for plants in the *Acanthopanax* genus.

**Table 2:** Amounts of the target components in the different *Acanthopanax* species.

Compound	ASCa	ASFa	ADAa	ADFa	ASBa	ADDa	AGa	AKa	ASSa	ATSa
eleutheroside B	82.76 ± 0.04	1.69 ± 0.04	2.00 ± 0.10	0.80 ± 0.02	1.08 ± 0.05	1.93 ± 0.02	3.26 ± 0.11	1.40 ± 0.02	0.64 ± 0.03	3.00 ± 0.04
eleutheroside E	73.89 ± 0.07	100.45 ± 0.37	134.37 ± 1.43	72.04 ± 0.60	20.86 ± 0.18	108.94 ± 0.31	3.44 ± 0.02	3.88 ± 0.04	22.03 ± 0.14	1.94 ± 0.01
chiisanoside	23.77 ± 0.34	22.40 ± 0.28	41.30 ± 0.52	20.04 ± 0.48	111.93 ± 1.40	32.72 ± 0.48	11.42 ± 0.35	15.07 ± 0.63	185.08 ± 7.49	13.81 ± 0.47
Sesamin	4.66 ± 0.09	20.91 ± 0.95	16.32 ± 0.08	28.20 ± 1.39	3.66 ± 0.17	12.4 ± 0.32	2.59 ± 0.11	21.84 ± 0.18	15.91 ± 0.38	N.D
Total	185.07 ± 0.53	145.45 ± 1.64	193.99 ± 2.13	121.09 ± 2.48	137.53 ± 1.81	156.00 ± 1.13	20.71 ± 0.58	42.18 ± 0.88	223.66 ± 8.04	18.75 ± 0.52

a Amounts are shown as μg/g.

N.D., not detected.

## 4 Conclusions

We developed an RP-HPLC-UV-IPAD method combining UV detection and IPAD detection to simultaneously analyze eleutheroside B, eleutheroside E, chiisanoside, and sesamin. This is the first report of an RP-HPLC-UV-IPAD method for determining the content of four different target components in the root bark of various *Acanthopanax* species. Our UV-IPAD method enabled the first high-sensitivity analysis of *Acanthopanax* root bark. As a novel attempt to analyze *Acanthopanax* species, the developed approach is believed to be helpful as a tool for establishing appropriate content criteria for *Acanthopanax* species.

## Declaration of competing interest

The authors have no conflicting financial interests to declare.

## Appendix A Supplementary data

supplemental Table 2 supplemental Table 2

supple figure 1 supple figure 1
