# Supplementary material for: Simultaneous quantification method for eleutheroside B, eleutheroside E, chiisanoside, and sesamin using reverse-phase high-performance liquid chromatography coupled with ultraviolet detection and integrated pulsed amperometric detection
Source: Heliyon. 2023 Jan 3;9(1):e12684. doi: 10.1016/j.heliyon.2022.e12684 (PMC9852659; doi:10.1016/j.heliyon.2022.e12684)
Supplement: supplemental Table 1 [file mmc1.pptx]

## Slide 1
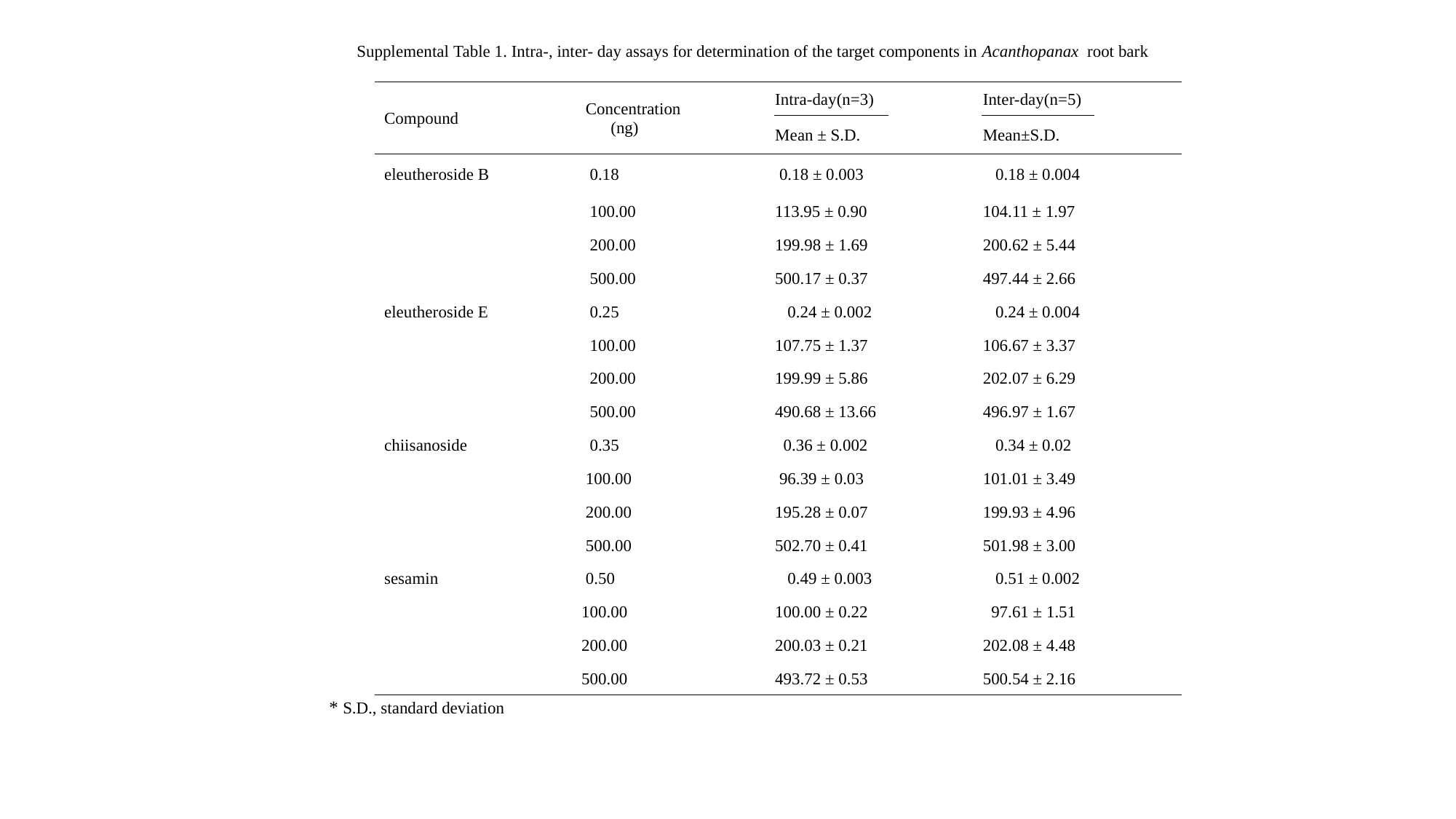

Supplemental Table 1. Intra-, inter- day assays for determination of the target components in Acanthopanax root bark
| Compound | Concentration (ng) | Intra-day(n=3) | | Inter-day(n=5) | |
| --- | --- | --- | --- | --- | --- |
| | | Mean ± S.D. | | Mean±S.D. | |
| eleutheroside B | 0.18 | 0.18 ± 0.003 | | 0.18 ± 0.004 | |
| | 100.00 | 113.95 ± 0.90 | | 104.11 ± 1.97 | |
| | 200.00 | 199.98 ± 1.69 | | 200.62 ± 5.44 | |
| | 500.00 | 500.17 ± 0.37 | | 497.44 ± 2.66 | |
| eleutheroside E | 0.25 | 0.24 ± 0.002 | | 0.24 ± 0.004 | |
| | 100.00 | 107.75 ± 1.37 | | 106.67 ± 3.37 | |
| | 200.00 | 199.99 ± 5.86 | | 202.07 ± 6.29 | |
| | 500.00 | 490.68 ± 13.66 | | 496.97 ± 1.67 | |
| chiisanoside | 0.35 | 0.36 ± 0.002 | | 0.34 ± 0.02 | |
| | 100.00 | 96.39 ± 0.03 | | 101.01 ± 3.49 | |
| | 200.00 | 195.28 ± 0.07 | | 199.93 ± 4.96 | |
| | 500.00 | 502.70 ± 0.41 | | 501.98 ± 3.00 | |
| sesamin | 0.50 | 0.49 ± 0.003 | | 0.51 ± 0.002 | |
| | 100.00 | 100.00 ± 0.22 | | 97.61 ± 1.51 | |
| | 200.00 | 200.03 ± 0.21 | | 202.08 ± 4.48 | |
| | 500.00 | 493.72 ± 0.53 | | 500.54 ± 2.16 | |
* S.D., standard deviation
