# Supplementary material for: Simultaneous quantification method for eleutheroside B, eleutheroside E, chiisanoside, and sesamin using reverse-phase high-performance liquid chromatography coupled with ultraviolet detection and integrated pulsed amperometric detection
Source: Heliyon. 2023 Jan 3;9(1):e12684. doi: 10.1016/j.heliyon.2022.e12684 (PMC9852659; doi:10.1016/j.heliyon.2022.e12684)
Supplement: supplemental Table 2 [file mmc2.pptx]

## Slide 1
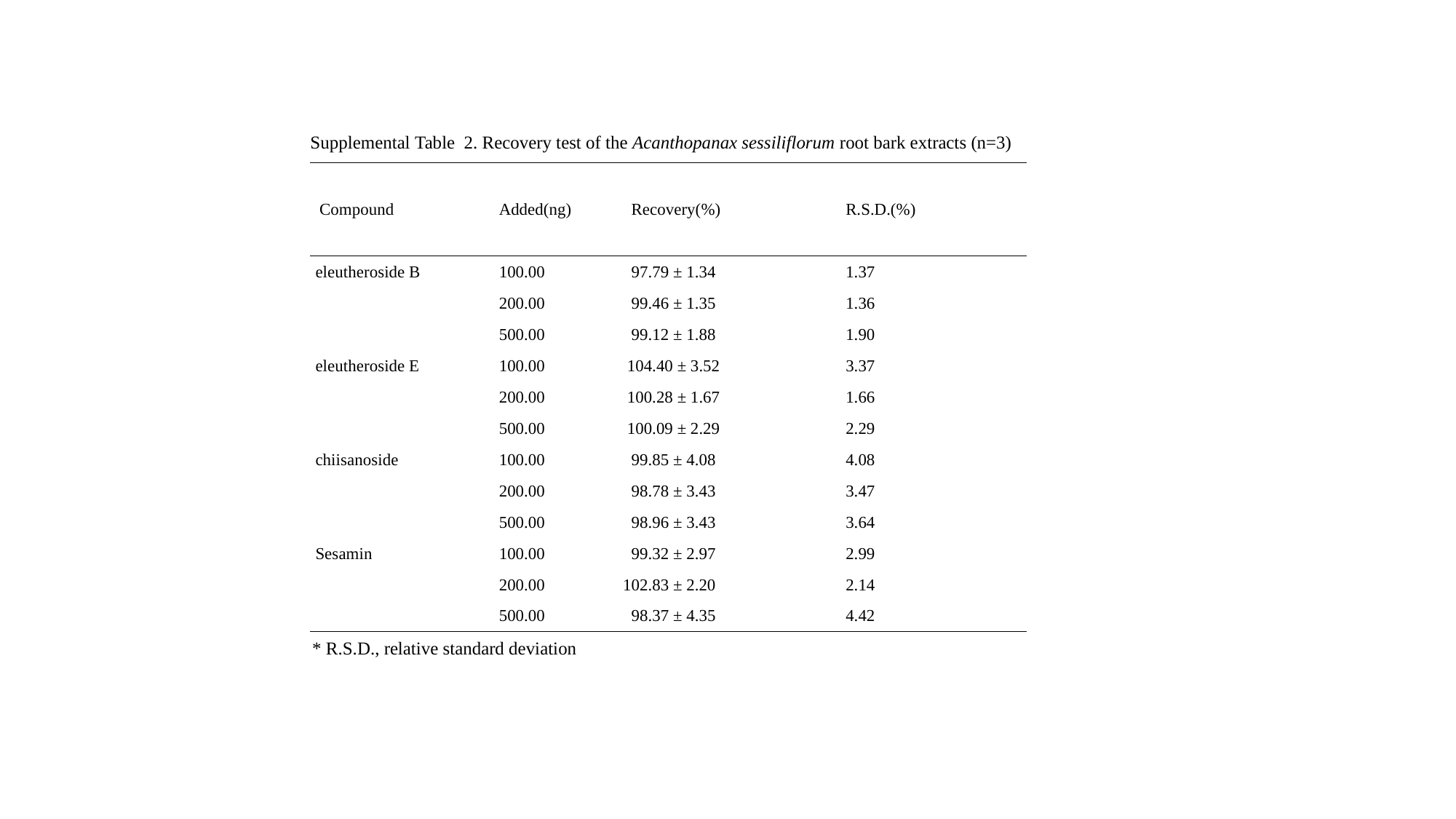

Supplemental Table 2. Recovery test of the Acanthopanax sessiliflorum root bark extracts (n=3)
| Compound | Added(ng) | Recovery(%) | R.S.D.(%) |
| --- | --- | --- | --- |
| eleutheroside B | 100.00 | 97.79 ± 1.34 | 1.37 |
| | 200.00 | 99.46 ± 1.35 | 1.36 |
| | 500.00 | 99.12 ± 1.88 | 1.90 |
| eleutheroside E | 100.00 | 104.40 ± 3.52 | 3.37 |
| | 200.00 | 100.28 ± 1.67 | 1.66 |
| | 500.00 | 100.09 ± 2.29 | 2.29 |
| chiisanoside | 100.00 | 99.85 ± 4.08 | 4.08 |
| | 200.00 | 98.78 ± 3.43 | 3.47 |
| | 500.00 | 98.96 ± 3.43 | 3.64 |
| Sesamin | 100.00 | 99.32 ± 2.97 | 2.99 |
| | 200.00 | 102.83 ± 2.20 | 2.14 |
| | 500.00 | 98.37 ± 4.35 | 4.42 |
* R.S.D., relative standard deviation
