# Supplementary material for: Simultaneous quantification method for eleutheroside B, eleutheroside E, chiisanoside, and sesamin using reverse-phase high-performance liquid chromatography coupled with ultraviolet detection and integrated pulsed amperometric detection
Source: Heliyon. 2023 Jan 3;9(1):e12684. doi: 10.1016/j.heliyon.2022.e12684 (PMC9852659; doi:10.1016/j.heliyon.2022.e12684)
Supplement: supple figure 1 [file mmc3.pptx]

## Slide 1
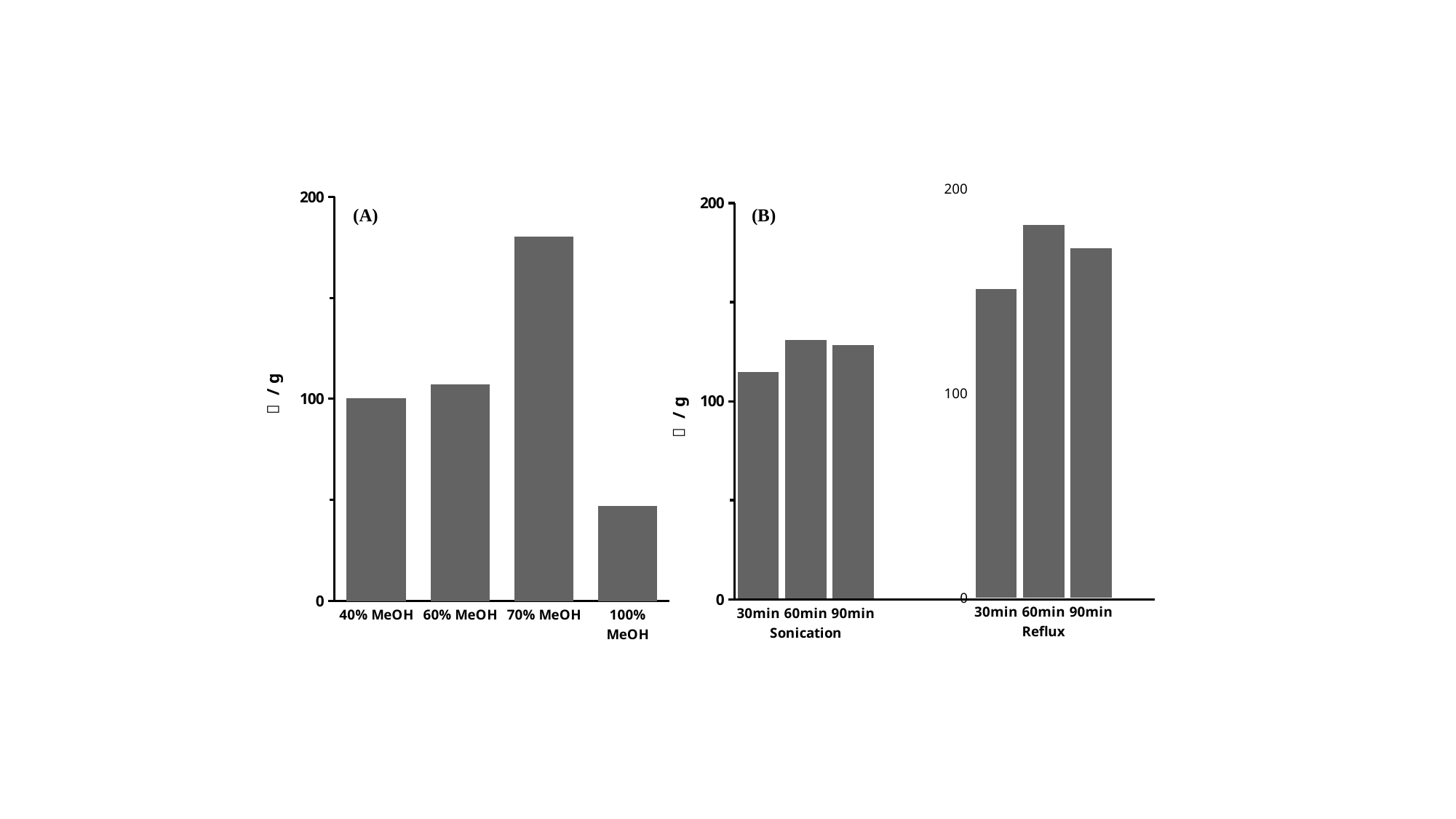

### Chart: Total content
| Category | Total content |
|---|---|
| 30min | 151.25715031372596 |
| 60min | 182.37509592808897 |
| 90min | 171.13628614252315 |
### Chart
| Category | Total content |
|---|---|
| 30min | 114.83189652678227 |
| 60min | 131.00456211957106 |
| 90min | 128.35744050686822 |(B)
### Chart
| Category | Total content |
|---|---|
| 40% MeOH | 100.46385860485552 |
| 60% MeOH | 107.14556979059046 |
| 70% MeOH | 180.14124114279247 |
| 100% MeOH | 46.85824698246937 |(A)
